# Mapping the Patient Experience in a Pediatric Hemophilia Unit: Our Patient Journey

**DOI:** 10.3390/jcm13206235

**Published:** 2024-10-18

**Authors:** Rubén Berrueco, Nuria Caballero, Mónica López-Tierling, Cristina Benedicto, Cristina González-Anleo, Natalia Rodríguez-Nieva, David Nadal, Joan Vinyets, Mercedes Jabalera

**Affiliations:** 1 Pediatric Hematology Department, Hospital Sant Joan de Déu, Sant Joan de Déu 2, 08950 Esplugues de Llobregat, Spaincristina.benedicto@sjd.es (C.B.); 2 Institut de Recerca Sant Joan de Déu de Barcelona (IRSJD), Santa Rosa 39-57, 08950 Esplugues de Llobregat, Spain; 3 National Institute of Biomedical Investigation in Rare Disorders (CIBER ER), Instituto de Salud Carlos III, 28029 Madrid, Spain; 4 Quality and Patient Experience Department, Hospital Sant Joan de Déu, Sant Joan de Déu 2, 08950 Esplugues de Llobregat, Spain; 5 Pharmacy Department, Hospital Sant Joan de Déu, Sant Joan de Déu 2, 08950 Esplugues de Llobregat, Spain; 6 Pediatric Rehabilitation Department, Hospital Sant Joan de Déu, Sant Joan de Déu 2, 08950 Esplugues de Llobregat, Spain

**Keywords:** hemophilia, children, patient journey, patient experience, human-centered design

## Abstract

**Background**: Hemophilia is a rare X-linked bleeding disorder. Prophylaxis has improved outcomes, but there are still unmet needs to be addressed. The aim of this study was to develop a patient journey in pediatric patients with hemophilia, a visual tool that illustrates patients’ relationship with the healthcare provider through time useful for identifying patient needs, potential concerns (“pain points”), and gaps in care. **Methods**: qualitative study in a pediatric hemophilia unit using a human-centered design methodology. First stage: discover and empathize: (a) semi-structured interviews to patients/families and stakeholders; (b) observation techniques (“shadowing”) to patients/families and professionals. Second stage: analyzing the collected information to create the patient journey. **Results**: A preliminary “clinical journey” was built using information from eight interviews with professionals from the interdisciplinary hemophilia team. Interviews with patient association representatives, 13 patients/families, and six “shadowing” techniques with patients and professionals were used to compare the “clinical journey” with the patient’s reported experience. Main “pain points” were detected before diagnosis, at diagnosis, during assimilation, at treatment initiation, during training, and when patients start asking about their condition. The empowerment process was detected as a potential moment to improve patient/family experiences. **Conclusions**: The patient journey helps to better understand patient/family experiences related to the disease in different scenarios. Caregivers and patient learning and empowerment processes are significant moments where the interdisciplinary team should focus to offer valuable solutions to improve outcomes. Further research is required in this area, particularly empirical research to amend or confirm the suggested patient journey.

## 1. Introduction

Congenital coagulopathies include a wide range of coagulation disorders with variable severity and risk of bleeding. Hemophilia A and B are rare X-linked bleeding disorders caused by deficiencies in factor VIII (FVIII) and factor IX (FIX), respectively [1]. People with hemophilia (PwH) may suffer from spontaneous or traumatic bleeding episodes that usually affect joints and muscles [1]. Recurrent hemarthrosis led to progressive joint destruction that has been related to a worsening of patient health-related quality of life (HRQoL) [2]. To mitigate this, prophylaxis has been established as the standard of care in PwH and severe bleeding phenotypes. This consists of the regular administration of therapeutic products to prevent hemorrhages while allowing patients to lead active lives and achieve an HRQoL comparable to that of non-hemophiliac individuals [1]. Prophylaxis is especially relevant in children, as early treatment is associated with better joint health status [1,3,4].

Research has dramatically improved the treatment landscape in hemophilia, but there still are unmet needs for patients and caregivers related to drug administration, adherence, inhibitor development, surgeries, or concomitant hemorrhages that can make treatment challenging. Most of the studies in this field have focused on clinical assessment of hemorrhage-related outcomes such as annualized bleed rate, joint health status, or HRQoL, and disease burden questionaries [5,6,7]. However, during the last decade, different patient-centered outcome measures have appeared to better understand qualitative and quantitative data such as treatment preferences or treatment impact on the patient goals [8]. Nevertheless, there is still a lack of information regarding relevant needs from the perspectives of patients and caregivers.

Human-centered design is a problem-solving methodology that emphasizes working with patients, understanding their problems, and creating solutions focused on real needs. It is divided into four stages. During stage 1, real needs are analyzed and discovered through observations and interviews; in stage 2, data are analyzed to identify points of improvement from observation to define action focuses and guide ideation. In stage 3, co-design sessions are used to look for ideas and possible solutions aimed at improving outcomes. Finally, in stage 4, pilot tests are conducted to validate possible improvements before launching the final solution [9,10,11]. The patient journey map is a tool developed at the end of the second stage that visually shows the relationship between the patient and the healthcare provider over time while providing information of internal and external factors that have an impact over the patient flow within the organization [9]. It helps to assess patient experience, to understand their needs and potential concerns, and to plan treatment or improve the transition processes lived through infancy and adolescence.

There are some publications in pediatric patients with other chronic conditions [12,13], but apart from a study focused on the treatment decision-making and gene therapy for hemophiliacs [14], there is scarce information of the patient experience in children with hemophilia. The aim of this work was to develop a pediatric patient journey through a human-centered design process in this setting, a visual tool that illustrates patients’ experience and their relationship with the healthcare provider through time useful for identifying patient unmet needs, potential concerns (“pain points”), and gaps in care.

## 2. Materials and Methods

A qualitative study using a human-centered design methodology to explore the pediatric patients’ and caregivers’ perspectives on the hemophilia patient journey was performed at a tertiary pediatric university hospital. It involved the multidisciplinary hemophilia unit team, researchers from the hospital’s patient experience team, pediatric patients <18 years of age with severe hemophilia A and B and their families/caregivers, and other stakeholders. No control group was considered for this project. There were no previous relationships between researchers from the patient experience team and the patients/caregivers or the pediatric multidisciplinary team, which helped them to provide an external overview on the bidirectional relationship between families and patients and health professionals.

Semi-structured interviews were designed specifically for each participating group: health professionals, patients older than 8 years of age, caregivers, and other stakeholders (Catalan hemophilia association) and had a duration of 30–90 min. Interviews were recorded and analyzed after transcription (Supplementary Table S1).

**Figure 1:**
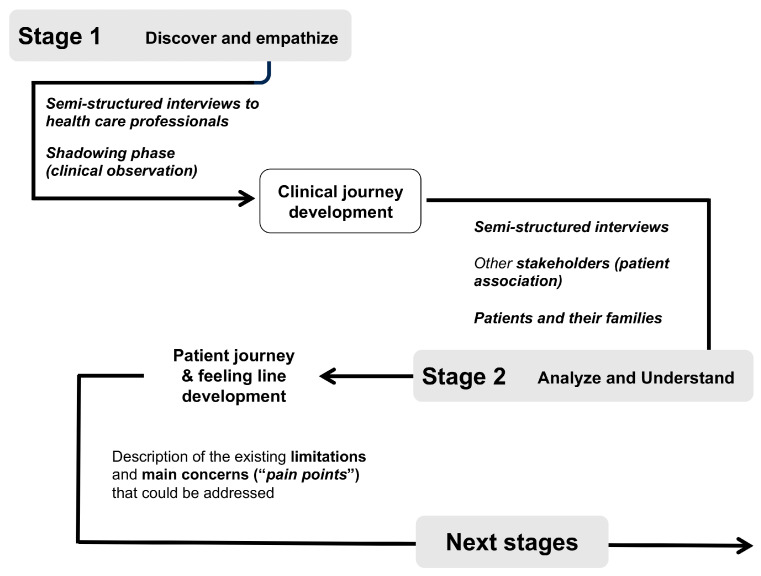
Main stages of the human-centered design process performed at the center.

The study was divided into two main stages (Figure 1).

### 2.1. First Stage: Discover and Empathize

- Health professionals’ semi-structured interviews to learn about the disease and the Hemophilia Unit at the hospital. Interviews covered seven main areas: general framework about professional dedication to hemophilia; hemophilia patient typology; characteristics of the relationship and communication with patient/caregivers through time; hemophilia information shared with patients/caregivers; patient/caregiver empowerment; patient/caregiver training for treatment administration and disease-related problem management; research expectations.
- Shadowing phase to incorporate observations made by researchers from the patient experience team during clinical appointments and nurse training sessions. Researchers acted as observers with the aim of evaluating the experience of patients and caregivers and analyzing their interaction with the health professionals. Researchers from the patient experience department are experts in performing this type of study and have been previously trained not to interfere with the patient or the health professionals at any time to avoid deviating them from their natural behavior under these circumstances.

At this point, researchers used all the collected information to create a preliminary health patient process called “clinical journey,” aimed to serve as a base to better understand hemophilia before designing interviews for patients and caregivers. This document included main disease stages and a list of well-known patient concerns defined as “pain points”.

- Semi-structured interviews with other stakeholders involved in the health care and empowerment of hemophilia pediatric patients to evaluate different points of view and detect weaknesses, strengths, and improvement opportunities. The interview structure was similar to those performed to health professionals.
- Semi-structured in-depth interviews with pediatric hemophilia patients and their families to obtain information regarding their experience in the Hemophilia Unit and regarding HRQoL and health literacy. Inclusion criteria: patients 1–18 years of age diagnosed with severe hemophilia receiving prophylactic treatment. In the case of patients less than 8 years of age or/and with mental disturbances, interviews were performed with the parents. The interdisciplinary team helped to identify patients and families who met the inclusion criteria, informed them of the study, and invited them to participate voluntarily. Participants were selected according to their disease status and different family conditions, such as age (<6 years old, 6–12 years old, >12 years old), inhibitor history (yes/no), family history (affected siblings), diagnosis due to severe bleeding (central nervous system bleeding), place of residence (more or less than 50 km), distance to the unit from their homes (more or less of 60 min), ethnicity, and family economic background.
- Semi-structured in-depth interviews with pediatric hemophilia patients also included participant and family observation at home.

### 2.2. Second Stage: Discover and Empathize

- First analysis of all the information derived from interviews, shadowing, and deep observation of the previous stage. Data underwent qualitative content analysis. Interviews were transcribed, and information was divided and categorized in different main areas while the patient journey was built. After analyzing the collected information during interviews with patients, families, and other stakeholders, researchers used the clinical journey as a base to expand it with other identified stages not previously detected. Its aim was to show all the processes that patients and caregivers need to face during time according to the age, the disease status, or possible complications of hemophilia, among others. Researchers also generated a feeling line to better understand patients and caregivers’ experience through time.
- Description of the existing limitations as well as the potential moments to improve patients and caregivers’ experiences.

Ethical issues were considered during our research. No ethical approval was sought as all collected data were anonymous and all the contributors joined on a voluntary basis. Moreover, all the participants signed an informed consent and a confidentiality agreement (caregivers gave permission for <18-year-old patients) and were free to withdraw from the project at any time. There were no payments for participants.

## 3. Results

A total of eight healthcare professionals and 14 patients/caregivers participated in the study (Supplementary Table S2). Collected information from a total of eight interviews with healthcare professionals and data from the shadowing phase was used to create a preliminary health patient process called the clinical journey (Table 1) that included five main domains (Section 3.1.1, Section 3.1.2, Section 3.1.3, Section 3.1.4 and Section 3.1.5). The main verbatims used can be seen in Supplementary Table S3.

### 3.1. Preliminary Clinical Journey

#### 3.1.1. Diagnosis

- Time for understanding and adjustment (for caregivers): Healthcare professionals individualize the information they share with caregivers according to the needs they detect. They are uncertain about the best moment to explain some details.

#### 3.1.2. Treatment Initiation and Training Program

- Overprotection: The stress following the diagnosis leads some families to overprotect patients, limiting their activities of daily life. Health professionals think that this may have negative effects on children’s self-esteem.
- Risk of getting used to being treated exclusively at the hospital: The training process is performed to guarantee that caregivers will be able to administer treatment at home. During this period, families establish a close relationship with the team, which makes them feel calm and peaceful. If families get used to the fact that the disease is only controlled at the hospital, it can make the training process more difficult.

#### 3.1.3. Follow-Up

- Lack of awareness in the correct use of treatment: Caregivers experience the benefits from prophylaxis. As patients have very few or even no bleeds, they sometimes think that their children are no longer in danger, which leads them to make inappropriate use of treatment.
- Need for support from peers: families and patients find great support from sharing their experiences with peers.
- Self-awareness in the disease: Patients grow up with hemophilia. They need to accept it and be aware of what it means to live with it. This process is different for each patient, as well as how or when they decide to talk about it in their environment.

#### 3.1.4. Teenager Training

- Carelessness and lack of self-awareness: Teenagers who have not experienced any bleeds may develop a lack of self-awareness of the disease. Education regarding the risks of living with hemophilia and treatment administration is crucial.
- Lack of delegation from caregivers: Adolescence is cherished by new roles and challenges for the families. Parents must delegate the responsibility of healthcare to their children while they experience the loss of the disease-controlling feeling they had.

#### 3.1.5. Transition to Adult Hospital

- Fear to change: Hospital dependency leads to an intense bond between professionals and families that can derive in some difficulties during the transition to the adult hemophilia unit.

### 3.2. Patient Journey

**Figure 2:**
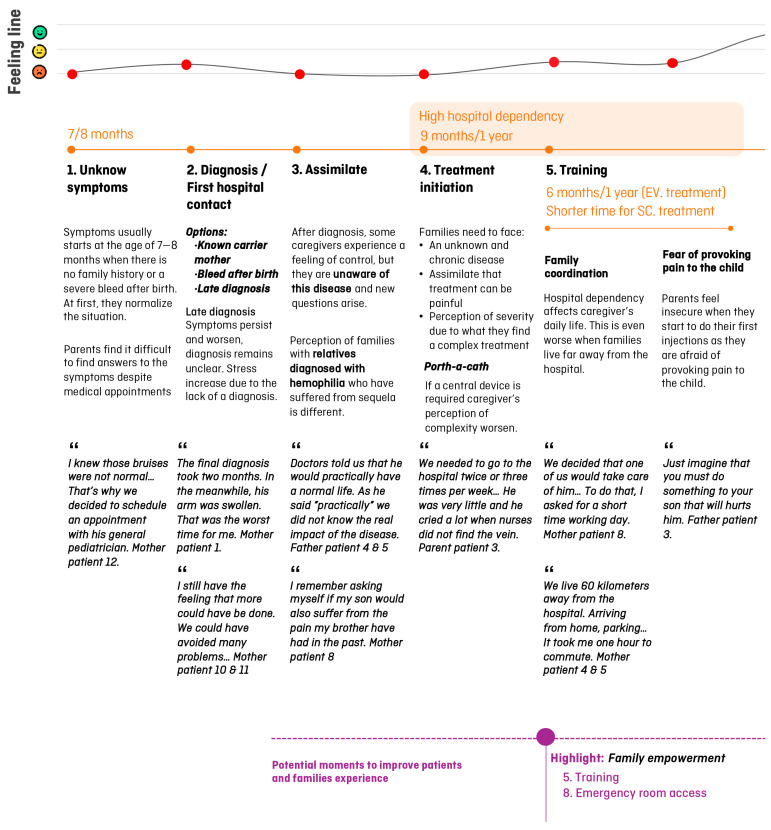
Patient journey in pediatric patients with hemophilia in Sant Joan de Déu Hospital (domains 1 to 5). Main conclusions are summarized below each domain. Verbatims are written in italics after quotes. Purple circles show the potential moments to improve patients and caregivers’ experiences. Feeling line express patients and caregivers’ experience through time (green: good; yellow: moderate; red: bad).

**Figure 3:**
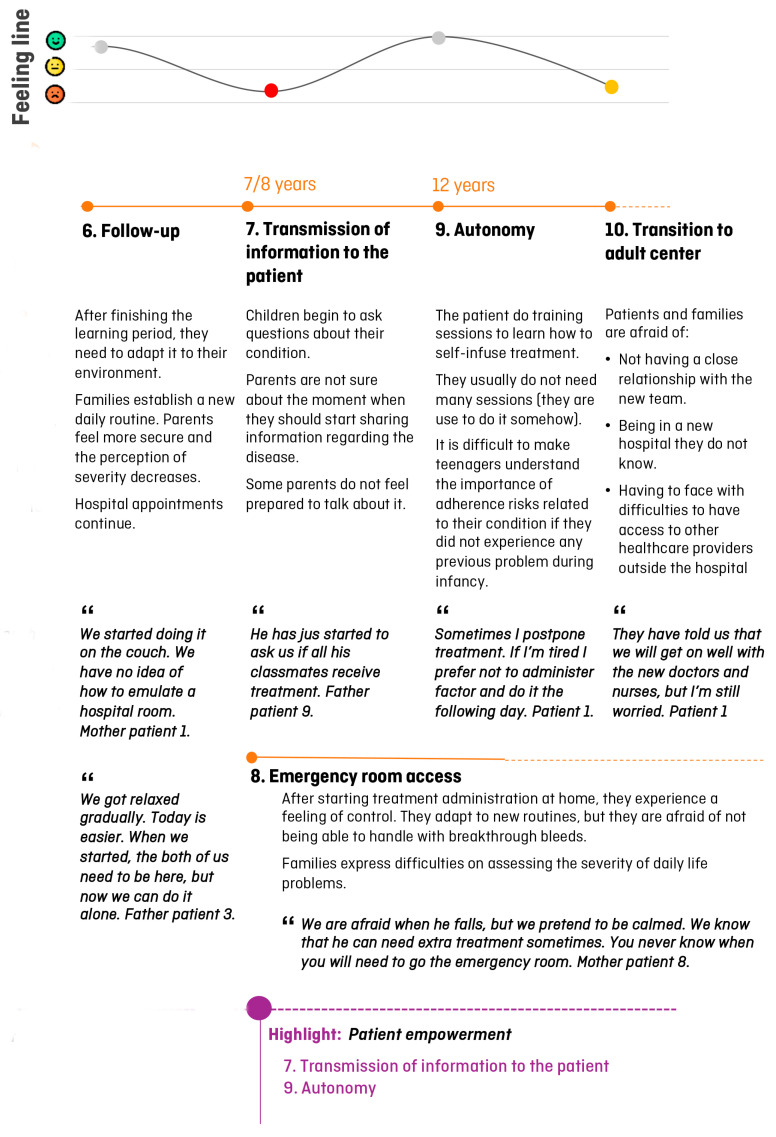
Patient journey in pediatric patients with hemophilia in Sant Joan de Déu Hospital (domains 6 to 10). Main conclusions are summarized below each domain. Verbatims are written in italics after quotes. Purple circles show the potential moments to improve patients and caregivers’ experiences. Feeling line express patients and caregivers’ experience through time (green: good; yellow: moderate; red: bad).

Once the clinical journey was created, researchers performed two semi-structured interviews with other stakeholders (family associations and a specialized school in hemophilia) and a total of 13 semi-structured interviews with patients/caregivers (Supplementary Table S2). Information collected was analyzed to create the patient journey and the feeling line (Figure 2 and Figure 3), which included a total of 10 domains (Section 3.2.1, Section 3.2.2, Section 3.2.3, Section 3.2.4, Section 3.2.5, Section 3.2.6, Section 3.2.7, Section 3.2.8, Section 3.2.9 and Section 3.2.10).

#### 3.2.1. Unknown Symptomatology

- Management of recurrent symptoms: When patients are not diagnosed during pregnancy (due to family history) or during the perinatal period, symptoms usually appear during the first 7–8 months of age. Families often normalize these problems. However, recurrent symptomatology leads them to seek answers at different medical appointments. It is not unusual for parents to be accused of handling their child too roughly to explain bruises.

“*When he started teething, he bled a lot, but as he is my first and only child, I thought it was normal*”.

(Mother patient 1)

#### 3.2.2. Diagnosis/First Hospital Admission

- Late diagnosis: Symptoms persist, diagnosis remains unclear, and bruises and bleeds appear after minor impacts. Fear, doubts, uncertainty, and stress increase during this period due to the lack of a definitive diagnosis.

“*When he was 3 months old, we noticed that his ankle was swollen. An X-ray was made and we were told that nothing was broken. It should improve by itself…*”.

(Mother patient 1)

“*He had a lot of bruises everywhere. Any time we had an appointment with the general pediatrician, I couldn’t stop thinking that social services would remove my son from my custody*”.

(Mother patient 6)

#### 3.2.3. Assimilation

- Unawareness of the disease: Diagnosis is accompanied by a feeling of control in those families who experienced uncertainties due to unknown symptomatology. However, new questions arise at this point. They do not know what hemophilia is, how it is treated, if their child will suffer pain, or if it will affect their HRQoL in the future. The lack of knowledge leads families to research, trying to understand and learn more about the disease. They receive information at the center, but they also search online, as they think it will provide them with a greater sense of control over the disease. Since information is poorly controlled, this has the potential to trigger more fear among caregivers.

“*Knowing the diagnosis was shocking… We were told that he would need medication for his whole life…*”.

(Mother patient 2)

- Ignorance regarding the family history: Sometimes, families have/had a relative diagnosed with hemophilia. Some do not have contact with this person and are unaware of the condition. However, others are very concerned, as they know the potential impact on the HRQoL.

“*I’m a carrier. I usually have easy bruises everywhere… For me, it was completely natural that my son also had bruises, but of course, I had no clue that this was the cause*”.

(Mother patient 3)

#### 3.2.4. Treatment Initiation

- Complex treatment: Children usually start prophylaxis around 9–12 months. Both options (subcutaneous or intravenous) are painful. Families not only need to face an unknown and chronic disease but also accept the fact that their child will continue experiencing pain. This feeling increases caregivers’ perception regarding the severity and complexity of the condition, which might be even worse when patients need a central venous device for treatment administration.

“*Learning to use the Port-a-cath was challenging […]. We came to the hospital three times per week to control that everything was ok*”.

(Mother patient 2)

#### 3.2.5. Training

Healthcare professionals have designed an individualized training program to empower caregivers to administer treatment at home. Intravenous administration training usually lasts between 6 and 12 months and has two main phases. First, parents undergo training in preparing treatment, locating veins, the correct position to do the injection, how to pick up the needle, and how to insert it through the skin. When they are prepared, parents start to administer treatment to their child. Families need to face different problems during this time:

- Family coordination to cope with the hospital dependency: Families may need hospital appointments once, twice, or three times per week. Hospital dependency affects their daily lives, especially when families live far from the hospital.

“*We had to go to the hospital twice a week… it was hard to combine work with this responsibility […]. We had to modify our timetable…*”.

(Father patient 3)

- Fear of provoking pain to their child: Clinical nurse specialists monitor the progress of caregivers’ learning process and encourage them to initiate puncture procedures at the hospital. Parents feel insecure when they start to do it, but once they feel more confident, families usually propose to start doing it at home. During this phase, parents are concerned about causing pain to their child.

“*To puncture is easy, but do it to your son is very difficult… We were always afraid of hurting him*”.

(Father Patient 7)

#### 3.2.6. Follow-Up

- Adjustment to home administration: When families complete the learning process, their sense of control increases. However, they must adapt the acquired knowledge to their household environment. Furthermore, they need to determine the optimal timing for administration. Nurses provide support, but the process remains challenging.

“*When we failed to administer the treatment the first time, we had to modify our position and that was very stressful for me. It was hard because I know that I passed my anxiety on to my son*”.

(Mother patient 13)

- Recovering daily life: Periodic prophylaxis administration at home enables families to establish a new daily routine, marking a crucial moment. Parents feel more secure managing the disease, and the perception of severity and complexity decreases.

“*We always do it very carefully. When we started to do it at home, we were extremely cautious, but now it’s just a routine*”.

(Mother patient 2)

#### 3.2.7. Transmission of Information to the Patient

- Knowledge transmission from parents to children: Around the age of 7–8 years, children begin to ask questions about their condition. Having grown up with hemophilia, they have normalized it. However, when they compare to their peers who do not receive treatment, they quickly realize they are different. Parents are not sure about when they should start sharing information regarding the disease and what to say. They are afraid of conveying overly serious information that might make their children feel inferior or trigger a rejection of the disease.

“*He thinks that everyone needs punctures… I know that he will soon realize that he is the only one who need it in his class. I am aware that we must talk about it naturally, but I feel it’s likely this can be frustrating for him… It’s easy to say “this is like wearing glasses”, but, at the end, it’s not the same…*”.

(Father patient 3)

#### 3.2.8. Emergency Room Access

- Dealing with problems: Once home treatment administration is started, families experience a feeling of better control. They easily adapt to new routines, but the fear of not being able to handle injuries or breakthrough bleeds persists. They express difficulties in assessing the severity of specific problems, such as what happens after a simple fall or a minor injury. Some families do not rely on the medical attention they receive outside the hospital, which makes things more difficult if they live far away.

“*We live in a small village. That is an important handicap because sometimes we must go to the hospital just because there is no access to a general pediatrician anytime. Moreover, when they realize he is “the child with hemophilia”, they always refer me to the hospital no matter what*”.

(Mother Patient 7)

#### 3.2.9. Autonomy

- Disease self-management: The perception of patients who never, or almost never, have experienced any significant problem related to hemophilia is that they only require periodic treatment. Sometimes, they do not understand the importance of adherence or the risks related to their condition. These facts led them to avoid health professionals’ recommendations, especially regarding self-treatment administration.

“*I live with my grandmother. If she doesn’t remind me that I have to put the treatment, I usually forget it. It’s tiring need to do it (administering treatment). As I feel fine, I don’t think I need to administer treatment so many times…*”.

(Patient observation)

#### 3.2.10. Transition to Adult Hospital

- Fear of change: When patients are around 16 years old, they start thinking about their referral to an adult hemophilia unit. Doubts are regarding the relationship they will establish with the new team and the access to health care.

“*Sometimes they tell us they are worried about the transition. However, they usually have the chance to know the new team in many of the association meetings. We think it helps a lot*”.

(Patient association representative)

## 4. Discussion

Human-centered design helps organizations better understand the problems patients might experience during healthcare assistance and how to address them [9]. During the first stage of our study, the multidisciplinary team created a journey based on five domains. After incorporating patient and caregiver experiences, additional relevant concerns such as the uncertainties secondary to the unknown symptomatology experience until final diagnosis, the initiation of treatment, the caregiver empowerment, as well as the transmission of information to children (when they are old enough to ask questions about the condition) were revealed. This information was used to build a patient journey that could be used as a basis for care in pediatric hemophilia units as well as induce more qualitative research.

Chronic diseases such as hemophilia have an important impact on the family [15,16]. Not surprisingly, the participation of caregivers in the development of our pediatric patient journey highlighted that the main concerns were related to uncertainties surrounding the disease, as previously described [17,18]. Family history of hemophilia is present in some cases, but this is not the most frequent situation in our center. Thus, exploring previous experiences related to symptoms preceding diagnosis is important to better understand caregivers’ feelings. It can be understandable that families tend to avoid those health professionals who failed in the initial diagnosis, but we usually insist on the importance of maintaining a close relationship with primary care teams, who should be responsible for non-hemophilia healthcare during infancy.

Diagnosis led to transient relief in the feeling line, but it was quickly followed by assimilation, a relevant pain point for families. They not only need to learn about the disease but also understand the main characteristics of substitutive and non-substitutive treatments if they want to have an active role in a shared decision process with the multidisciplinary team, including a possible participation in a clinical trial [3]. Education is crucial to do so. It should start at an early stage and continue over time. In fact, family support must be tailored during the caregiver’s educational program, ensuring that they are adequately trained to self-administer treatment at home [19]. However, it is remarkable that uncertainty persists over time instead of prophylaxis. Caregivers fear about the future and possible long-term sequelae, more pronounced in specific scenarios such as those patients diagnosed in the context of a cerebral hemorrhage that will need a specific approach [18,20].

There are critical transitions that need to be addressed. First, children receiving treatment need to learn to treat themselves, a gradual learning process previously described [19] that our work has related to an improvement of patients’ feelings. Afterwards, transition to an adult hospital/unit was another relevant pain point. At this moment, and according to literature [21], feeling line decreased.

From the parents’ perspective, they first become caregivers when they learn to treat their children. Later on, they will need to rely on the teenager’s capacity to self-care. In the meantime, our work also highlighted that most parents fear the moment when children start to ask questions about their condition, around the age of 7–8 years. It is known that receiving support from an early childhood on some ideas, such as the usefulness of prophylactic treatment to live a normal family life, can be very helpful [18,19,21]. However, other parents and caregivers’ needs should be evaluated periodically to understand those difficulties and challenges they face over time.

For us, it is relevant to acknowledge the feeling of uncertainty that cherish caregivers through time. Its importance relies on the fact that some studies described that the use of positive coping strategies may ameliorate anxiety, depression, and other negative self-reported psychological outcomes associated with uncertainty, which suggests a role for intervention [17]. The term free-mind hemophilia is a recently described concept aimed at examining the absence of psychological burden and of permanent thoughts about the disease and its complications in people with hemophilia [22]. After building this pediatric patient journey, we propose the definition of a “caregiver free-mind hemophilia mind”.

Patient journey is a tool that should be read carefully and individualized for each patient, family, hemophilia center, and country [9]. Accordingly, feeling line evaluation could be misleading for unexperienced readers. It is not related to the quality of healthcare professionals’ work; it exclusively represents how the patient and caregivers feel while facing disease-related problems through time. Moreover, the patient journey should be dynamic and remain under continuous revision/reconstruction. For instance, a few years ago, a pediatric patient journey would have likely identified two main patient archetypes based on the presence of inhibitors. Nowadays, new drugs such as emicizumab, useful for prophylaxis in patients with hemophilia A, with and without inhibitors [23], make these differences no longer relevant. In fact, we know that the disruption of new substitutive, non-substitutive treatments will have an impact on our patient journey.

This work has some strengths and limitations. The number of patients included in the study was low, but patient selection took into account different characteristics such as age, family history of hemophilia, or the distance to the unit. Moreover, interviews with patients and caregivers were performed at their homes, which improved our understanding of each family context. However, we understand that our patient journey could not be easily extrapolated to other units. Moreover, it does not include patients with mild bleeding phenotypes or other congenital coagulopathies. The aim of this work was not to focus on female carriers. However, information gathered from mothers and sisters of participants in this research will be used for another ongoing project. Finally, as the patient journey extends throughout all life stages for PwH, this work needs to be continued in the adult hemophilia center.

## 5. Conclusions

The patient journey is a tool that is useful for better understanding patient experience through time as well as identifying unmet needs related to healthcare assistance. Our work highlights that uncertainties are stressful for caregivers and persist beyond the diagnosis. Empowerment and learning processes are potential pain points where intervention could be useful for improving patients’ and families’ experiences.

## Figures and Tables

**Table 1:** Main domains of the preliminary clinical journey.

Preliminary Clinical Journey				
Section 3.1.1	Section 3.1.2	Section 3.1.3	Section 3.1.4	Section 3.1.5
Time for understanding and adjustment (for caregivers).	Overprotection. Risk of getting used to being treated exclusively at the hospital.	Lack of awareness in the correct use of treatment. Need for support from peers. Self-awareness in the disease.	Carelessness and lack of self-awareness. Lack of delegation from caregivers.	Fear to change.

## Data Availability

The raw data supporting the conclusions of this article will be made available by the authors on request.

## Supplementary Materials

The following supporting information can be downloaded at: https://www.mdpi.com/article/10.3390/jcm13206235/s1, Table S1: Scheme used for semi-structured interviews. Table S2: Main characteristics of participant involved in the study; Table S3: Verbatims used for creating the clinical journey.
