# Supplementary material for: Mapping the Patient Experience in a Pediatric Hemophilia Unit: Our Patient Journey
Source: J Clin Med. 2024 Oct 18;13(20):6235. doi: 10.3390/jcm13206235 (PMC11508278; doi:10.3390/jcm13206235)
Supplement: Supplementary file 1 [file jcm-13-06235-s001.zip › jcm-3247190-supplementary.pdf]

Supplementary Materials

Supplementary Table S1. Scheme used for semi-structured interviews.

	**Professionals**	**Patients/Caregivers**
**Context**	-Role in the hospital<br>-Daily life at the unit<br>-Organization and coordination of the hemophilia unit at the hospital and with external stakeholders	-For how long they are being treated at the unit<br>-Understanding their daily life in relationship with hemophilia<br>-Relationship with other healthcare providers different to the hemophilia unit
**Patient typology**	-Type of patients<br>-Type of treatments<br>-How is diagnosed<br>-Treatment depending on symptoms<br>-Impact of the disease/treatment on quality of life<br>-Treatment decision (who/when)<br>-Treatment administration (hospital/home)<br>-Patient needs	-Diagnosis<br>-Treatment (current and previous)<br>-How diagnosis was made<br>-How treatment was initiated<br>-Impact of the treatment and the disease on the quality of life<br>-Participation on treatment decision<br>-Treatment administration through time (hospital/home)<br>-Patient/caregivers needs
**Patient and families/caregiver appointments**	-Type, periodicity, duration of appointments<br>-Who performs each appointment<br>-Who is coming with the patient<br>-Interaction with the patient (without the parent/caregiver)<br>-Interaction with the family<br>-Role of families and patients during each appointment<br>-Difficulties<br>-Recurrent doubts and questions<br>-Possible improvements	-Type, periodicity, duration of appointments<br>-Who performs each appointment<br>-Who is usually going with the child<br>-Interaction with the healthcare professional<br>-Interaction with the team<br>-What is their role during the appointments<br>-Difficulties they find<br>-Recurrent doubts and questions<br>-Possible improvements
**Information and knowledge of the disease**	-Information offered to the family after diagnosis<br>-Information offered to the patient/family afterwards<br>-How and type of information given<br>-Who offers all this information<br>-Other possible sources of information (including social media)<br>-Possible supporting groups for families and patients<br>-How to improve the way communication to the family is done<br>-There is any feedback from the family?	-Type of information received after diagnosis<br>-Type of information received over the time<br>-Characteristics of the information<br>-From whom they received it.<br>-Other sources where they have look for information<br>-Do they participate in some type of patient/family supporting group?<br>-How information could be improved?<br>-Have they had the opportunity to give any feedback?
**Empowerment and decision making**	-Treatment options<br>-Do families have time to make a decision?<br>-Problems to administrate a specific treatment.	-Do the know treatment options?<br>-Do they have time to make a decision regarding treatment?<br>-What problems they find for treatment administration?

	-Do patients actively ask for new treatments?<br>-Patient/family implication on the treatment decision making<br>-How to improve their empowerment?	-Do they make questions to the professionals regarding it?<br>-Do they have a real implication on the treatment decision making?<br>-How they think this can improve?
**Learning process, training and living with the disease**	-Objectives for training (treatment self-administration).<br>-Who receives training<br>-When, how, to whom it is started.<br>-Patient/family needs during the process<br>-Duration of training<br>-Professionals who participate<br>-When it finishes<br>-Questions and recurrent doubts<br>-Living with the disease<br>-How to manage urgent situations	-Description of training (since it started to it finished): when started, duration, who participated, from whom they received it, difficulties, expectations, questions, fears…<br>-Living with the disease: problems they need to face with, recurrent questions and doubts, fears.<br>-How they ask for help<br>-How to manage urgent situations.
**Project expectations**	-Expectations about what will be the final result and how this project will help them and patients/families.	-Expectations about how this project will help them/other families.

Supplementary Table S2. Main characteristics of participant involved in the study. *Interviews in patients less than 8 years of age or in those with mental impairments were performed to the parents.

**Patients interviewed**					
**Code**	**Age**	**Inhibitor history**	**Family history**	**Bleeding symptoms at diagnosis**	**Distance to the Unit**
Patient 1	16 years	No	No	Joint bleed	< 50 Km
Patient 3	3 years*	No	No	Joint bleed	< 50 Km
Patient 7	5 years*	Yes	No	Mild bleed at birth	> 50 Km
Patient 8	11 years	No	Yes	Bruises	< 50 Km
Patient 10	18 years*	Yes	Yes	Cerebral hemorrhage	< 50 Km
Patient 11	16 years	Yes	Yes	Brother patient 9	< 50 Km
Patient 13	13 years	No	No	Bruises	< 50 Km
**Patients interviewed and observed (shadowing)**					
Patient 2	6 years*	Yes	No	Cerebral hemorrhage	< 50 Km
Patient 4	12 years	No	Yes	Mild bleed at birth	> 50 Km
Patient 5	5 years*	No	Yes	Brother patient 4	> 50 Km
Patient 6	2 years*	No	No	Bruises	> 50 Km
Patient 9	5 years*	No	Yes	Bruises	> 50 Km
Patient 12	1.5 years*	Yes	No	Cerebral hemorrhage	< 50 Km
**Patients observed (shadowing)**					
Patient 14	17 years	Yes	No	Bruises	< 50 Km
**Healthcare professionals**					
**Code**		**Role**		**Time from recruitment**	
Healthcare professional 1		Hematologist		10 years	
Healthcare professional 2		Hematologist		4 years	
Healthcare professional 3		Rehabilitation doctor		5 years	
Healthcare professional 4		Clinical nurse specialist		8 years	
Healthcare professional 5		Clinical nurse specialist		5 years	
Healthcare professional 6		Clinical trial nurse		8 years	
Healthcare professional 7		Pharmacist		2 years	
Healthcare professional 8		Social worker		10 years	

Supplementary Table S3. Verbatims used for creating the clinical journey.

**3.1.1. Diagnosis**
**Time for understanding and adjustment (for carergivers)**<br>· *"Will there ever be a good moment to tell them?" (Health care professional (HC) 1, hematologist)*
**3.1.2 Treatment initiation and treatment program**
**Overprotection**<br>· *"There's a boy whose mum is very overprotective. He wants to learn how to inject himself, but his mum won't let him. She also won't let him go skiing with his school friends. The mum is just scared, but his son is frustrated. What can we do in this situation?" (HC-4, clinical nurse specialist)*<br>· *"It's necessary to show them that their son needs to enjoy his childhood like other kids do. He needs to try things, to feel free. We are here to help and keep an eye on him. I think that, deep down, the parents know they are too protective, but it's impossible for them not to act differently". (HC-3, rehabilitation doctor)*<br>**Risk of getting used to being treated exclusively at the hospital**<br>· *The mum explains that «I don't think I'll be able to put the injections as well as nurses do». She feels more secure coming to the hospital. (Participant observation, patient 12)*<br>· *"Sometimes, parents ask for social help to continue treatment at the center as much as possible." (HC-8, social worker)*
**3.1.3. Follow up**
**Lack of awareness in the correct use of treatment**<br>· *"I sometimes think that families are convinced that, regardless the problem, administering factor is always the solution. This isn't necessary true, but this attitude is often perceived during the appointments". (HC 1, hematologist)*<br>· *"Treatment has an analgesic effect. Parents have learnt to administer factor when the child is in pain, but this is not always necessary. To administer an extra dose is not a big deal for them, but parents need to be aware of this situation". (HC-3, rehabilitation doctor).*<br>· *"We explain (to the caregivers and the patients) that they need to administer the factor three mornings per week but, sometimes, we realize that they are administering it in the afternoons… They usually tell us the truth, but from time to time, I've the feeling that they're not explaining things they are doing wrong." (HC 2, hematologist)*<br>· *"It is recommended that medication is refrigerated at all times. If I tell them that treatment can be stored out of the fridge for some time, I think they will take it easy and won't pay as much attention to this important feature…" (HC 7, pharmacist)*<br>**Need for support from peers**<br>· *"As a doctor, I know that I'm not aware of relevant things… I had a meeting with a group of mothers in the Catalan hemophilia association. A mother told there that, when his child was not receiving prophylaxis, she used to wake up every single night to check if her son was still breathing. She decided to do it after talking to me after diagnosis, but I'm sure I never recommended her to do something like that." (HC 1, hematologist).*<br>· *"I will never be able to put myself in the shoes of a hemophiliac's mother. I can talk to them, but it is not comparable when two of them share their own experiences." (HC 5, clinical nurse specialist)*<br>**Self-awareness in the disease**<br>· *"Hemophilia is an invisible disease. People don't know that a patient suffers from it unless they are told. However, some patients are used to say «I'm a hemophiliac» as an excuse to do whatever they want (HC1, hematologist)*<br>· *"Some children don't want to explain their disease to other peers. We tell them that they don't need to explain it to everyone, but it is also important to talk about it to those friends close to them. It is important that they know it in case they have an accident or a bleeding episode." (HC6, clinical trial nurse)*

3.1.4. Teenager training
**Carelessness and lack of self-awareness**<br>· *"It is not easy to make that a teenager understand that he needs to do physiotherapy… If he is doing fine and, from his point of view, he has a normal life, he won't do anything." (HC3, rehabilitation doctor)*<br>· *"It is necessary that they understand the importance of adherence to treatment even though they are not suffering from bleeding. It serves as a long-term preventive measure (HC1, hematologist)*<br>· *Regarding factor administration, a teenager patient says that he already knows what to do, and how to do it. He doesn't want to be bothered by nurses, who call him to check if he's self-administering treatment. However, he admits that if his grandmother doesn't remind him that he needs to administer the factor, he usually forgets it. (Participant observation)*<br>**Lack of delegation from caregivers**<br>· *"There are two main challenges. On the one hand, it is necessary that the patient is willing to start self-administration. On the other, parents should be ready to allow this to happen. If they are overprotective, they won't want to take this step." (HC 5, clinical nurse specialist)*<br>· *"Patients learn to self-administer treatment very quickly. They have lived seeing it for many years… [ ]. Parents need to know that they must give their son the chance to do it by himself." (HC 4, clinical nurse specialist)*<br>· *"Whereas parents become experts in hemophilia, some children turn 18 knowing nothing about it." (HC1, hematologist).*
**3.1.5. Transition to adult hospital**
**Fear to change**<br>· *"Transition is not easy. Patients depend on us when we are empowering them to self-administer the treatment. When we invite them to do it by themselves at home, it seems as if we are abandoning them in some way, but it is not. We encourage them to do it at home because they need to be as much independents as they can before transition." (HC 5, clinical nurse specialist).*<br>· *"Our aim is to empower them to avoid that they will need help from us in the future but, at the same time, letting them know that we will always be here for them." (HC 4, clinical nurse specialist).*
